# Synophthalmia in a Holstein cross calf

**Published:** 2014

**Authors:** Hossein Nourani, Iraj Karimi, Hossein Rajabi Vardanjani

**Affiliations:** 1 *Department of Pathobiology, Faculty of Veterinary Medicine, Ferdowsi University of Mashhad, Mashhad, Iran; *; 2 *Department of Pathobiology, Faculty of Veterinary Medicine, Shahrekord University, Shahrekord, Iran; *; 3 *Department of Pharmacology, Faculty of Pharmacy, Student Research Committee of Ahvaz Jundishapur University of Medical Sciences, Ahvaz, Iran.*

**Keywords:** Craniofacial anomalies, Cyclopia, Holstein calf, Synophthalmia

## Abstract

Synophthalmia is a form of cyclopia, in which some elements of two eyes are fused and form a single eye in the middle region of the forehead. The head of a Holstein female calf born from a 5-year-old cow was referred to Department of Pathology, School of Veterinary Medicine, Shahrekord University due to multiple congenital anomalies. The calf had been slaughtered immediately after birth due to severe respiratory distress by the owner. The calf showed multiple birth defects, including synophthalmia, holoprosencephaly, absence of optic chiasma, hypoplastic maxilla, curved mandibles, arrhinia and dental pad agenesis. A normal tongue protruded from the defect and small oral cavity. To our knowledge, this particular combination of craniofacial defects has not been previously described in Holstein calf.

## Introduction

Cyclopia is the presence of a single median orbita that contains either a single eyeball, true cyclopia or incompletely fused eyeballs, synophthalmia.^1^

Over 50 years ago scientists demonstrated that holo-prosencephaly and the related craniofacial deformities, called ‘monkey face lamb disease’ were produced in lamb fetuses when pregnant ewes grazed on *Veratrum californicum *early in gestation. ^2^

In cow, cyclopia has been reported in a brown swiss cross calf,^1^ Friesian calf,^3^ German Fleckvieh calf,^4^ Hariana breed calf. ^5^

To the authors’ experiences, this particular combination of craniofacial defects has not been previously described in Holstein calf and this report describes macroscopic characteristics of a unique congenital abnormalities.

## Case Description

The head of a Holstein female calf born from a 5-year-old cow was referred to Department of Pathobiology, School of Veterinary Medicine, Shahrekord University, Iran due to multiple congenital anomalies. There was no record of disease, treatment and suspicious diet in the history of dam. The calf was slaughtered immediately after birth due to severe respiratory distress by the owner. The head was examined grossly and the anomalies recorded.

**Fig. 1:**
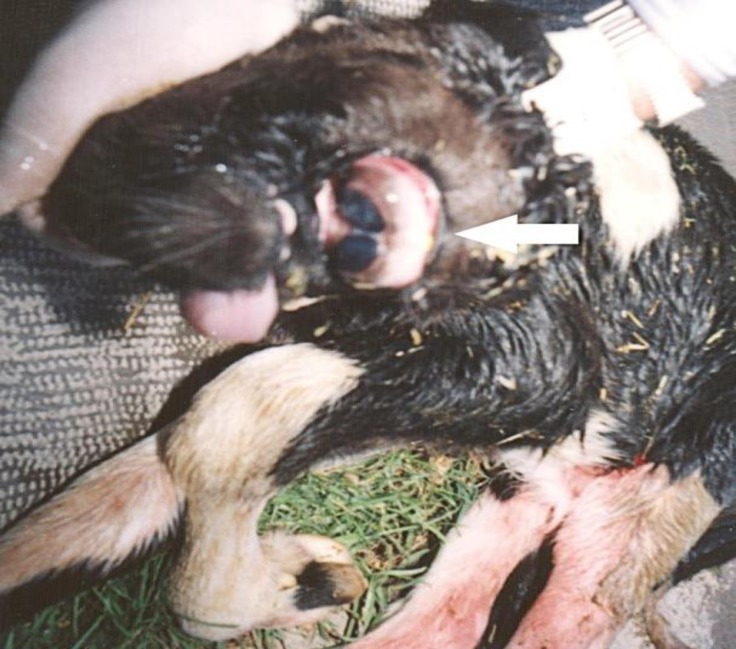
Synophthalmia. A single median orbita (arrow) with incompletely fused exophthalmic eyeballs.

**Fig. 2:**
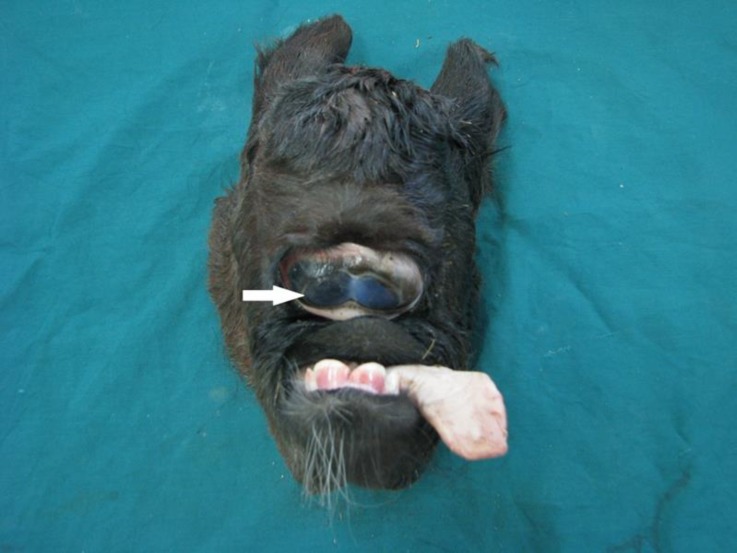
Synophthalmia (arrow), arrhinia and protrusion of tongue

**Fig. 3:**
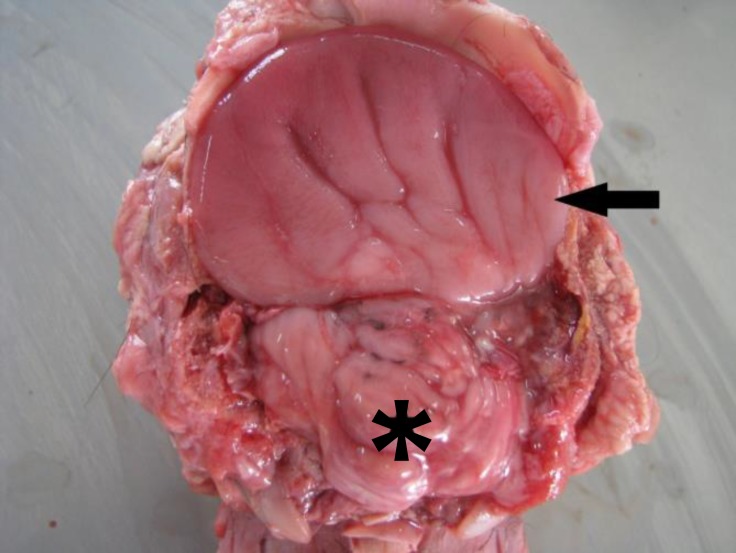
Dorsal view of holoprosencephaly. There are no cerebral hemispheres (arrow), Cerebellum (asterisk).

The most striking malformation was the presence of a single median orbita that contained incompletely fused exophthalmic eyeballs (Figs. 1 and 2). Duplication of anterior intraocular structures, such as lens and pupil were found but there was one optic nerve. The brain was primitive with no cerebral hemispheres (holoprosencephaly) and gray and white matters differentiation (Fig. 3). 

**Fig. 4:**
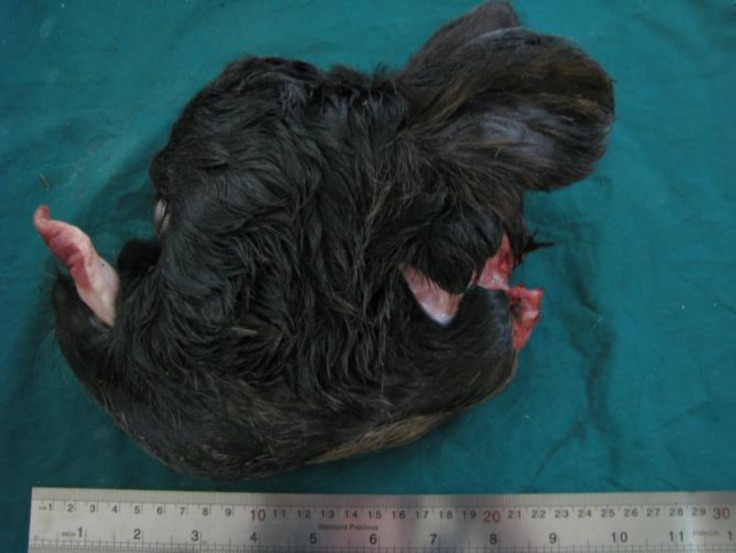
Lateral view of the head with strongly curved left mandible.

There was no optic chiasma. Other important defects included hypoplastic maxilla, curved mandibles, arrhinia and dental pad agenesis. A normal tongue was protruded from the defect and small oral cavity (Figs. 2 and 4).

## Discussion

In this case, the possible cause of the craniofacial defects could not be ascertained. In sheep and goat, craniofacial defects such as cyclopia or synophthalmia are caused by ingestion of plants of Veratrum species. These anomalies are varied from the extreme malformation, cyclopia to mildly deformed upper jaws.^6^

The toxins of *Veratrum californicum* were shown to be steroidal alkaloids, primarily cyclopamine.^6^ Cyclopamine is teratogenic and inhibits the hedgehog (hh) signal trans-duction pathway. Mouse embryos cultured in the presence of cyclopamine, showed cyclopia and the associated developmental brain defect holoprosencephaly.^7^ Cyclo-pamine-induced malformations in chick embryos are associated with interruption of sonic hedgehog (hh) mediated dorsoventral patterning of the neural tube and somites.^8^ Cyclopamine induced cyclopia is reported in rabbit embryos too.^6^

In humans, the synophthalmia is a result of neural plate misdevelopment syndrome involving the eye, brain, skull and face. It is well known that synophthalmia is due to heterogenous causes, most of which to chromosomal abnormalities.^9^

In the present case, nose, upper jaw and the cerebral hemispheres were absent. Similar anomalies have been reported by Minoru and Katsumi, 1999 in three calf cases of cyclopia.^10^

To our knowledge, this particular combination of craniofacial defects has not been previously described in Holstein calf.
